# Mirizzi Syndrome: an unexpected problem of cholelithiasis. Our experience with 27 cases

**DOI:** 10.1186/1477-7800-5-12

**Published:** 2008-05-21

**Authors:** Michael Safioleas, Michael Stamatakos, Panagiotis Safioleas, Anastasios Smyrnis, Constantinos Revenas, Constantinos Safioleas

**Affiliations:** 1 2nd Department of Propaedeutic Surgery, Medical School, University of Athens, Laiko General Hospital, Athens, Greece; 2 Department of Radiology, Laiko Hospital, Athens, Greece

## Abstract

**Purpose:**

Mirizzi syndrome is a rare complication of long standing cholelithiasis. The purpose of this study is to retrospectively estimate the diagnostic and treatment methods applied in patients with Mirizzi syndrome.

**Materials and methods:**

Our experience with 27 cases with Mirizzi syndrome is presented. They were diagnosed either by imaging techniques, or during surgical operation. All of the patients were managed surgically.

**Results:**

8 patients were diagnosed preoperatively and the rest intraoperatively. Morbidity rate after surgery was 18,5%, and mortality rate was zero. The patients presented free of symptoms three months after surgery during the follow-up.

**Conclusion:**

Mirizzi syndrome is rarely diagnosed preoperatively and US proved inadequate for this purpose. Surgery is the only therapy and usually provides additionally definitive diagnosis.

## Introduction

Mirizzi syndrome is an unusual complication of gallstone disease and occurs in approximately 1% of all patients with cholelithiasis [1]. The syndrome was first described in 1948 and is characterized by impaction of stones in the cystic duct or neck of the gallbladder, resulting in mechanical obstruction of the common hepatic duct and frequent clinical presentation of intermittent or constant jaundice [2]. The majority of cases are not identified preoperatively, despite the availability of modern imaging techniques.

Thus a constant vigilance during intraoperative dissection of Calot's triangle is required in order to avoid injury of the bile duct.

## Patients-methods and results

This is a retrospective study of 27 consecutive patients with Mirizzi syndrome in the last 20 years (1986–2005), treated at the 2^nd ^Department of Propaedeutic Surgery, Medical School, University of Athens. A total of 2,978 cholecystectomies were performed during this period. Among the 27 patients with Mirizzi syndrome, 21 patients (77,7%) had type I disease, 5 patients (18,5%) had type II disease and one patient (3,7%) had type II/III disease. The records of these 27 patients were reviewed for clinical symptoms, diagnostic methods, surgical procedures, complications and follow-up. The Csendes classification was followed to categorize the patients. For types II, III or IV when cholecystobiliary fistula existed, the classification was made intraoperatively. All patients were seen in the surgical department within 3 months from their discharge from the hospital and every 6 months thereafter for a mean period of five years (range: 3–13 years). Four patients were lost from follow-up 1–2 years after surgery. All were symptom free with normal liver function tests through the last follow-up visit. None of the patients with Mirizzi syndrome had previous hepatobiliary surgical intervention prior to diagnosis. Females outnumbered males by sixteen to eleven. The age of patients ranged from 48 years to 86 years. Upper abdominal pain, fever and jaundice were the most frequent symptoms and signs. The diagnosis of Mirizzi syndrome was achieved preoperatively in 8 patients (29,6%). The other 19 patients (70,3%) were diagnosed intraoperatively. For preoperative evaluation of the patients the technological advancements of the last three decades were employed.

Despite arguments in the literature about the usefulness of ultrasonography (US) in diagnosis, it proved to be of limited value, as in only 8 patients was US indicative for Mirizzi syndrome. In these 8 cases, the diagnosis was confirmed by endoscopic retrograde cholangiopancreatography (ERCP) in 6 cases, while by magnetic resonance cholangiopancreatography (MRCP) and percutaneus cholangiography (PC) the remaining two cases. The diagnostic value of the employed non-invasive imaging techniques in our series is shown in table 1. In our series dilated hepatic radicals were observed in 13 patients. One case of cholecystocholedochal fistula was identified in the preoperative work-up. All of the patients were operated on using, either a right subcostal incision (20 cases), or right paramedian incision (6 cases).

**Table 1:** Diagnostic value of the non invasive imaging techniques used in our patients with Mirizzi Syndrome.

**TECHNIQUE**	**N° PATIENTS**	**DIAGNOSTIC**	**%**
US	27	8	29.6
CT SCAN	6	0	0
ERCP	11	6	54.5
MRCP	2	1	50

Surgical treatment was applied in all patients with Mirizzi syndrome. In 21 patients with type I disease, partial cholecystectomy was performed in 15 cases and complete cholecystectomy in 6 cases. In 4 cases laparoscopic cholecystectomy was attempted but only in one case it was achieved. Among the six patients with cholecystobiliary fistula, in five patients with type II disease T-tube insertion from the fistula or directly to the common bile duct using the gallbladder as a pedicled graft in either case was performed.

**Figure 1:**
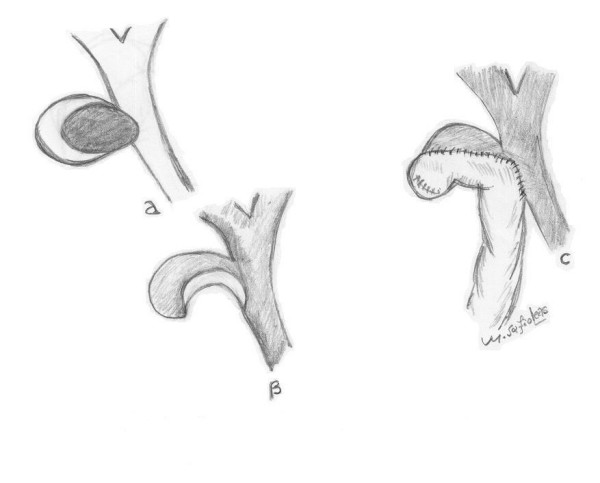
Schematic representation of the cholecysto-choledochal jejunostomy.

In the remaining patient with type II or III disease with a particular appearance of the fistula opening after extraction of the gallstone (photo) and considering the risk of postoperative biliary stricture increased, a Roux-en-Y cholecysto-choledochal-jejunostomy was carried out (Fig. 1). No bile leak was noted in any patient with cholecystobiliary fistula, whereas bile leakage was noted in 3 patients treated by partial cholecystectomy. Spontaneous stop of leaking was observed in both cases after 4 and 6 days respectively. In the remaining case biliary stenting on the 10^th ^postoperative day was necessary. All patients had a tube drain left in the subhepatic space that remained for 2 to 15 days. Furthermore, two patients developed wound infection necessitating open drainage. Overall morbidity was 18,5% (5 cases). The mortality rate in our series was zero. Histology did not reveal concomitant carcinoma of the gallbladder in any of the patients.

## Discussion

Mirizzi syndrome (MS) was described in 1948 as obstructive jaundice due to gallstone/s impacted in the cystic duct or Hartmann's pouch, compressing the common hepatic duct^2^. McSherry in 1982 suggested a subclassification of the Mirizzi syndrome into two types.

Type I regards the external compression of the common hepatic by a calculus in the cystic duct or Hartmann's pouch whereas, in type II the stone has eroded partially or completely into the common hepatic bile duct and a cholecystocholedochal fistula has resulted [3].

In 1989 a new classification of patients with Mirizzi syndrome and cholecystobiliary fistula was presented. Type I lesions are those with external compression of the common bile duct; in type II lesions a cholecystobiliary fistula is present with erosion of less than one third of the circumference of the bile duct; in type III lesions, the fistula involves up to two thirds of the duct circumference; and in type IV there is complete destruction of the bile duct [4].

Therefore, Mirizzi syndrome and cholecystobiliary fistula appear to be different, evolving stages of the same pathological condition; thus, it is reasonable that Lubbers proposes that the term Mirizzi syndrome could not be abandoned, as it is only the first stage of a more complete process [5].

Nevertheless, gallstone erosion into the common duct is a rare complication of cholelithiasis and is mainly the result of the long-standing gallstone disease. Cholecystocholedochal fistula is an important complication of Mirizzi syndrome but it occurs only in 1% of all biliary operations [1,4]. Clinical diagnosis of Mirizzi syndrome is difficult, since there are no pathognomonic patterns in presentation. Blood investigations are not very helpful. Diagnostically, ultrasound is the first screening method but may miss the presence of Mirizzi syndrome, as CT can also do. ERCP is able to confirm the diagnosis in 50% of cases. MRCP can be as good as ERCP in diagnosis and its ability to delineate details of biliary strictures, but its disadvantage compared to ERCP is its inability to confirm the presence of fistula and offer therapeutic stenting [6]. On the other hand, T_2 _sections can differentiate a neoplastic mass from an inflammatory one, which U/S or CT scan may not be capable of doing.

The association of Mirizzi syndrome and gallbladder carcinoma is also of interest; in such cases it is obvious that complex surgical procedures should be avoided [7,8]. However, despite all these modern diagnostic modalities, it is possible for the problem to become apparent only during operation [9]. Intraoperatively, the presence of Mirizzi syndrome can be suggested by the finding of intense adhesions between the gallbladder and the common hepatic duct in the area of Calot's triangle [10].

In fact, surgery is the treatment of choice for patients with diagnosis of Mirizzi syndrome. The surgical strategy includes complete removal of the gallbladder or partial cholecystectomy for MS type I, while various surgical approaches have been used for MS type II, where cholecystobiliary fistula is present and requires treatment.

The following approach is rather more accepted for type II. After the gallstone has been removed and partial cholecystectomy has been performed, the remaining gallbladder is used for choledochoplasty. A T-tube is introduced into the common hepatic duct above the repair site. Furthermore, in our department, in order to close the hole in the common bile duct, we have used, besides the gallbladder cuff, a pedicled graft of the round ligament of the liver [11,12]. Recently we have performed a Roux-en-Y cholecysto-choledochal-jejunostomy with favorable outcome in a patient with MS type II/III based on operative findings [13].

In our opinion, an 8–15 mm cuff of the gallbladder or round ligament is sufficient in order to close the defect in the common bile duct.

The role of laparoscopic approach in the treatment of Mirizzi syndrome remains controversial. Some authors consider the condition unsuitable for laparoscopic surgery since the inflammatory tissue in the area of Calot's triangle offers a high operative risk in dissection [6,14,15]. Other authors propose laparoscopic surgery in the treatment of Mirizzi syndrome [16-18]. We consider that in presence of cholecystocholedochal fistula conventional laparotomy is mandatory. Even in type I Mirizzi syndrome, laparoscopic surgery is not always feasible. Thus, in our series three operations with initial access by laparoscopy were converted to laparotomy due to major technical difficulties.

In high-risk patients suffering from MS, biliary drainage by endoscopic sphincterotomy and placement of a stent in the choledochal duct has been carried out [19]. Moreover placement of a nasobiliary catheter in conjunction with cholangioscopy for electrohydraulic lithotripsy has been reported [20,21].

Although the diagnostic imaging techniques have been perfected, preoperative diagnosis of MS is not an easy affair and continues to be a challenge for the surgeon. Therefore, even intraoperative precautious recognition of the condition and application of the appropriate surgical method according to the characteristics of each case will lead to successful treatment. In conclusion, it is important to identify patients with Mirizzi syndrome preoperatively but it seems even more important to consider its diagnosis during surgical dissection.

## Abbreviations

U/S: Ultrasonography; ERCP: Endoscopic retrograde cholangiopancreatography; MRCP: Magnetic resonance cholangiopancreatography; PC: Percutaneous cholangeography; MS: Mirizzi Syndrome; CT: Computed tomography

## Competing interests

The authors declare that they have no competing interests.

## Authors' contributions

M.Safi Surgeon who performed the operation and edited a part of the manuscript.

MSta who performed the operation and prepared the draft.

PS Literature search, revision of bibliography.

AS Surgeon who contributed to the performance of the operation.

CR Radiologist who made the diagnosis of the laboratory findings.

CS Literature search, revision of bibliography.

All authors have read and approved the final manuscript.
